# Non-Invasive Imaging of Acute Renal Allograft Rejection in Rats Using Small Animal ^18^F-FDG-PET

**DOI:** 10.1371/journal.pone.0005296

**Published:** 2009-04-24

**Authors:** Stefan Reuter, Uta Schnöckel, Rita Schröter, Otmar Schober, Hermann Pavenstädt, Michael Schäfers, Gert Gabriëls, Eberhard Schlatter

**Affiliations:** 1 Medizinische Klinik und Poliklinik D, Experimentelle Nephrologie, Universitätsklinikum Münster, Münster, Germany; 2 Klinik und Poliklinik für Nuklearmedizin, Universitätsklinikum Münster, Münster, Germany; L' Istituto di Biomedicina ed Immunologia Molecolare, Consiglio Nazionale delle Ricerche, Italy

## Abstract

**Background:**

At present, renal grafts are the most common solid organ transplants world-wide. Given the importance of renal transplantation and the limitation of available donor kidneys, detailed analysis of factors that affect transplant survival are important. Despite the introduction of new and effective immunosuppressive drugs, acute cellular graft rejection (AR) is still a major risk for graft survival. Nowadays, AR can only be definitively by renal biopsy. However, biopsies carry a risk of renal transplant injury and loss. Most important, they can not be performed in patients taking anticoagulant drugs.

**Methodology/Principal Findings:**

We present a non-invasive, entirely image-based method to assess AR in an allogeneic rat renal transplantation model using small animal positron emission tomography (PET) and ^18^F-fluorodeoxyglucose (FDG). 3 h after i.v. injection of 30 MBq FDG into adult uni-nephrectomized, allogeneically transplanted rats, tissue radioactivity of renal parenchyma was assessed *in vivo* by a small animal PET-scanner (post operative day (POD) 1,2,4, and 7) and post mortem dissection. The mean radioactivity (cps/mm^3^ tissue) as well as the percent injected dose (%ID) was compared between graft and native reference kidney. Results were confirmed by histological and autoradiographic analysis. Healthy rats, rats with acute CSA nephrotoxicity, with acute tubular necrosis, and syngeneically transplanted rats served as controls. FDG-uptake was significantly elevated only in allogeneic grafts from POD 1 on when compared to the native kidney (%ID graft POD 1: 0.54±0.06; POD 2: 0.58±0.12; POD 4: 0.81±0.06; POD 7: 0.77±0.1; CTR: 0.22±0.01, n = 3–28). Renal FDG-uptake *in vivo* correlated with the results obtained by micro-autoradiography and the degree of inflammatory infiltrates observed in histology.

**Conclusions/Significance:**

We propose that graft FDG-PET imaging is a new option to non-invasively, specifically, early detect, and follow-up acute renal rejection. This method is potentially useful to improve post-transplant rejection monitoring.

## Introduction

The number of patients necessitating treatment for end-stage renal disease, and therefore, the number of patients with renal transplants still increases [1]. At present, renal grafts are the most common solid organ transplants world-wide. Despite the introduction of new and effective immunosuppressive drugs, acute cellular graft rejection is still a major risk for graft survival. In a recently published follow-up analysis of over 28000 renal allograft recipients, more than 4000 patients experienced at least one episode of acute rejection (AR) within 3 years (21.6% experienced even more than one AR in the first postoperative year). 83.2% of these acute rejections occurred within the first 3 months post-transplantation [2]. Episodes of acute allograft rejection are a negative prognostic factor for the development of chronic renal allograft failure (CAF) and for long-term graft survival [2]–[4]. The impact of AR on chronic renal allograft failure rises, whereas the severity of episodes was identified as an independent risk factor [4], [5]. CAF remains the most common cause for death-censored graft-loss after renal transplantation [6], [7]. Post-transplant monitoring basically relies on monitoring of renal retention parameters, like creatinine and urea, in addition to the observation of non-specific symptoms frequently present in AR, i.e., proteinuria, oliguria, hypertension, graft tenderness, or peripheral edema. (Doppler-) Ultrasound examination can detect rejection-related irregularities in the graft perfusion, but its specificity and sensitivity for AR is limited, even when echo enhancers are applied [8], [9]. Furthermore, sensitivity and reliability of this method mainly depend on the investigators experience. Radionuclide-based methods for the measurement of renal function, i.e., TER-MAG-clearance, can disclose impairment of renal function, but might be of limited value, if acute tubular necrosis or cyclosporine A toxicity are involved [10], [11]. Invasive core needle biopsies are still the “gold-standard” in rejection diagnostics [12], [13]. Nevertheless, they are cumbersome to the patient and carry the risk of significant hematuria, arteriovenous fistulas, and graft loss [14], [15]. Notably, they can not be performed on patients taking anticoagulant drugs. Therefore, a non-invasive tool for specific detection of AR, which can be applied in the early phase of rejection, is desirable.

AR of renal grafts is characterized by recruitment of activated leukocytes into the transplant [16], [17], which is an integral part of the basic concept of the Banff-classification, a commonly used renal rejection score [12]. Activated leukocytes show a distinct accumulation of ^18^F-Fluor-Desoxy-Glucose (FDG) which can be measured by positron emission tomography (PET) [18]. FDG-PET is widely used for detection, staging and follow-up of inflammation or tumors, respectively [18], [19].

The aim of this study was to establish and validate an entirely image-based method for the early detection and follow-up of renal allograft rejection in rats in order to translate this approach to human patients in the future. It is based on high-resolution whole body small animal PET following i.v. injection of FDG. FDG-uptake of renal parenchyma was assessed in uni-nephrectomized, allogeneically kidney-transplanted animals (aTX) with, and additionally in native controls (CTR) and syngeneically transplanted (sTX) animals without rejection or impairment of renal function. Because acute cyclosporine A nephrotoxicity (CSA) is a reversible process in which renal function can recover if the drug administration is stopped at the early stage of the disease, differential diagnosis of this disease with other renal dysfunctions that occur after kidney transplantation is of interest. In particular, acute tubular necrosis (ATN) caused by ischemia/reperfusion injury during the transplantation procedure shows a similar clinical course. Therefore, we included two additional groups into the study. One group with acute CSA nephrotoxicity and another with renal ischemia/ reperfusion damage developing acute tubular necrosis (ATN). PET results *in vivo* were compared to FDG-activity of renal parenchyma in micro-autoradiography and in transplants to histological changes of acute renal rejection (glomerulitis, tubulitis, endothelialitis, and infiltration) *ex vivo*.

## Results

### Analysis of PET images and quantitative evaluation

**Figure 1:**
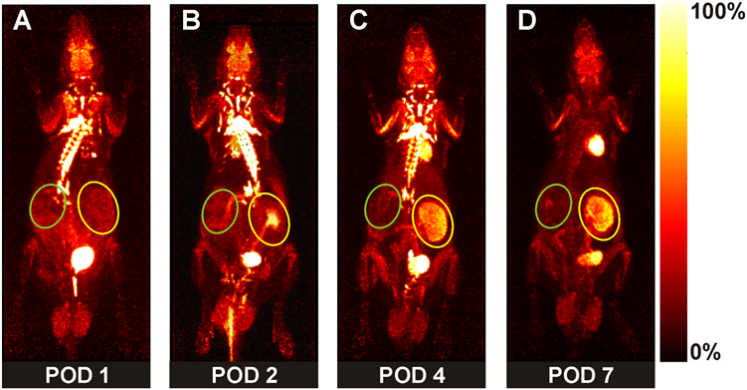
Representative PET-images of dynamic whole body acquisitions of a series of an allogeneically transplanted rat (aTX) (POD 1 (A), 2 (B), 4 (C), and 7 (D), after tail vein injection of 30 MBq FDG (maximum a posterior (MAP) projection, 180 min p.i.). While the parenchyma (yellow circle) of renal allograft accumulates FDG with a maximum on POD 4, the native kidney (green circle) does not show any accumulation at any time. Please note that the renal pelvis can contain eliminated FDG. Therefore, it was excluded from the measurements.

**Figure 2:**
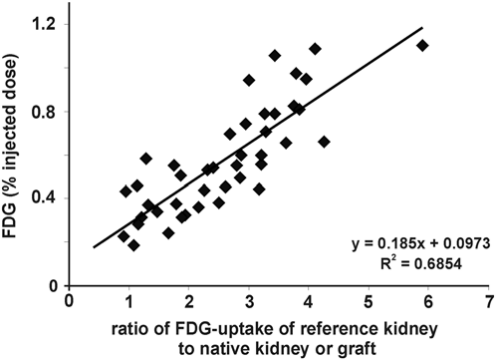
Correlation between the FDG uptake of graft kidneys expressed in percentage of the injected dose (%ID) and the ratio of FDG uptake of graft kidney to control kidney. A significant correlation of the ratio and the percent injected dose was found (r^2^ = 0.69).

**Figure 3:**
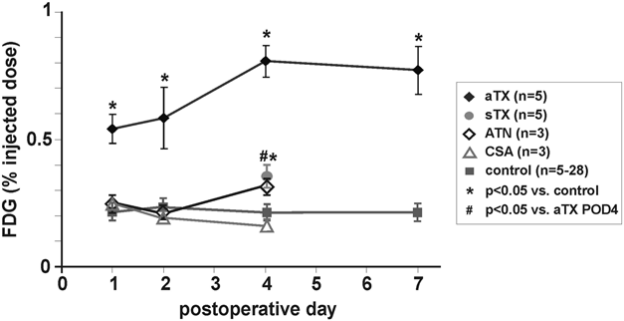
Detection of acute rejection by measurement of the %ID of FDG within renal allografts. Kidneys undergoing acute rejection showed a clearly increased FDG-uptake starting from the first postoperative day (POD 1) (0.54±0.06%, n = 5 vs. 0.22±0.02%, n = 13, in controls). The %ID of allogeneic transplants rose further until POD 4 (0.58±0.12%, n = 5, POD 2, and 0.81±0.06%, n = 5, POD 4), while that of native kidneys remained the same (0.2±0.03%, n = 5, day 4). Towards POD 7 the %ID of allografts remained stable (0.77±0.1%, n = 5). The %ID of syngeneic transplanted controls was 0.37±0.04% (n = 5, POD 4). This was not significantly different to controls but to aTX (p<0.05). The FDG uptake of kidneys with acute tubular necrosis (ATN: POD1: 0.25±0.02%, POD2: 0.21±0.04%, POD4: 0.31±0.02%, n = 3) or acute cyclosporine A toxicity (CSA: 0.24±0.03%, POD2: 0.19±0.01%, POD4: 0.16±0.01%, n = 3) was not significantly different to that of control kidneys.

In allografts undergoing AR, we detected a clearly elevated FDG-uptake starting on postoperative day 1 (POD) (Figure 1), when compared to the contralateral reference kidney (aTX/CTR = 2.74±0.96, n = 5). The allograft/control-ratio peaked on POD 4 (3.36±0.45, n = 5, Figure 1C). We conclude that early detection of AR by PET analysis is possible, when the FDG-uptake of control and grafted kidney is compared. Nevertheless, patients usually lack a control kidney, thus a measurement independent of a reference organ would be preferable. Liver is often referred to as a reference organ. However, PET referring to FDG-uptake of liver needs fasting of subjects, which limits serial investigation in small animals having undergone surgery. Therefore, we used the previously calculated FDG-uptake ratios of the reference (native) kidney to their allogeneic, syngeneic transplant or to their second native kidney (healthy controls), respectively. Then, we assessed the percentage of FDG-uptake (% injected dose, %ID) within the parenchyma of the graft or the remaining native kidney, respectively. FDG-uptake of grafted kidneys (in %ID) showed a significant correlation to the ratio of FDG-uptake of graft to control kidney (R^2^ = 0.69, Figure 2). Thus, %ID replacing the ratio graft/control analogously reflects the “rejection status” (aTX>sTX>control) of the measured kidney. %ID of aTX grafts (0.54±0.06%, n = 5 vs. CTR: 0.25±0.03%, n = 25) increased starting from POD 1 (Figure 3) and peaked towards POD 4 (0.58±0.12% POD 2 and 0.8±0.06% POD 4, n = 5), while %ID of the control kidneys remained constantly low (0.2±0.02% on day 4, n = 10). From POD 4 to POD 7 no significant change in %ID of allografts and controls was observed (0.77±0.1%, n = 5, vs. 0.21±0.02% in CTR, n = 5). When comparing the %ID of syngeneic transplanted controls at the maximum activity time point in aTX at POD 4, %ID in sTX was significantly lower than in aTX and only slightly higher than the %ID of native kidneys (0.37±0.02%, n = 5) (Figure 3). The FDG uptake of kidneys with acute tubular necrosis (ATN: POD1: 0.25±0.02%, POD2: 0.21±0.04%, POD4: 0.31±0.02%, n = 3) or acute cyclosporine A toxicity (CSA: 0.24±0.03%, POD2: 0.19±0.01%, POD4: 0.16±0.01%, n = 3) was not significantly different to that of control kidneys.

### Assessment of renal function by calculation of the renal fluoride clearance

**Figure 4:**
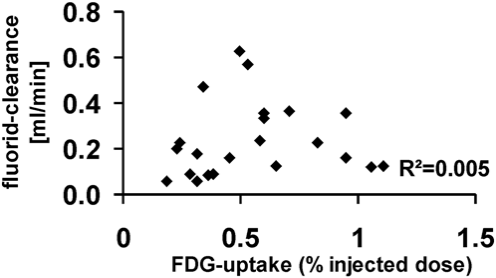
Relation of the ^18^F-fluoride clearance (ml/min) to the FDG uptake (%ID). No correlation of these two parameters was found (R^2^ = 0.005, n = 22).

We assessed renal ^18^F-fluoride clearance in a subgroup of animals to exclude decreased renal function as a potential cause for increased FDG-uptake into the renal parenchyma. FDG-uptake (%ID) did not correlate to ^18^F-fluoride-clearance (R^2^ = 0.005; Figure 4).

### Micro-autoradiographic quantification of FDG-uptake of renal parenchyma

**Figure 5:**
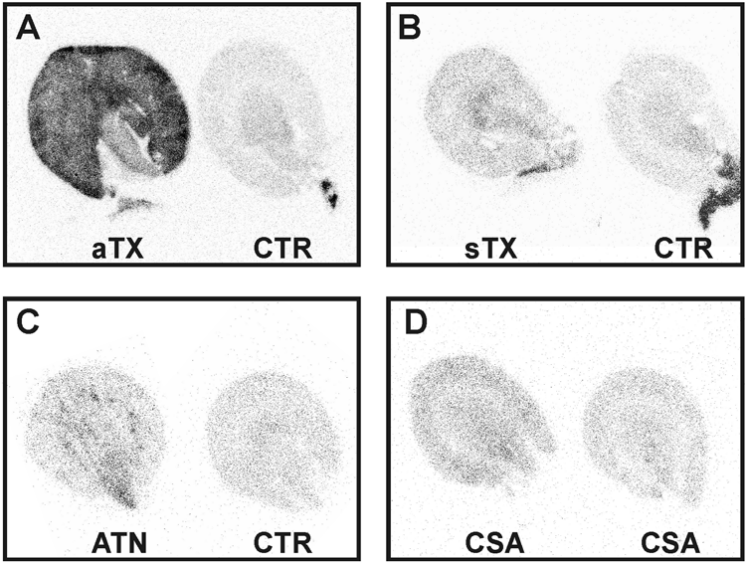
Signs of acute rejection, namely glomerulitis, tubulitis, endothelialitis, and graft infiltration, were found in the allograft group (aTX) starting from POD 1 (A). Nevertheless, graft integrity was confirmed on POD 1, 2, and 4. In contrast, histology of POD 7 showed the appearance of necrosis and hemorrhage within the allograft. No histological signs of rejection were found in native controls (leukocytes: 12±1/FOV, n = 18) or syngeneic transplants (sTX) (leukocytes: 26±4/FOV, n = 6). Leukocyte infiltration in kidneys with acute tubular necrosis (ATN, POD4, leukocytes: 17±3/FOV, n = 3) was moderate and emphasized in the outer medulla, while kidneys with acute cyclosporine toxicity (CSA) presented with only 7±1/FOV leukocytes (POD 4, n = 6). In allografts significant infiltration was found since POD 1 (B) (leukocytes: 77±2/FOV, all groups p<0.05 vs. sTX and CTR, n = 6). It aggravated on POD 2 (leukocytes: 98±12/FOV), peaked on POD 4 (leukocytes: 142±4/FOV), and was slightly lower on POD 7 (leukocytes: 115±2/FOV).

**Figure 6:**
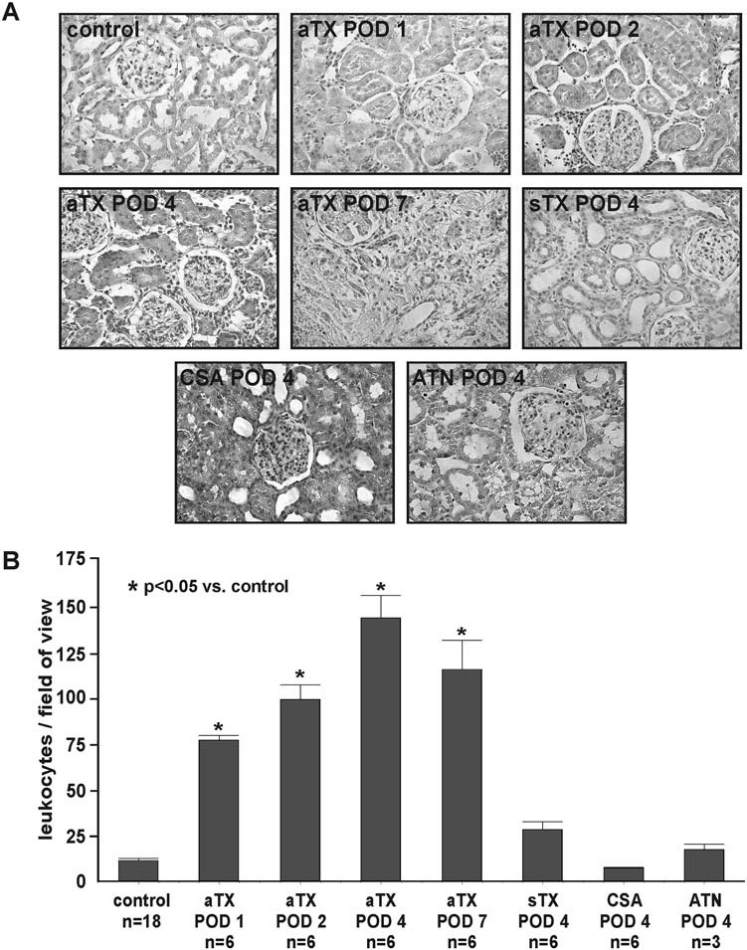
Representative micro-autoradiography of an allogeneically (aTX), a syngeneically transplanted kidney (sTX), a kidney with acute tubular necrosis (ATN), two kidneys with acute cyclosporine A toxicity (CSA), and control kidneys 180 min p.i. of 30 MBq FDG, 4 days after surgery or treatment, respectively. Control kidneys (A–C, right kidneys) showed low mean FDG-uptake/mm^2^ in the mid-coronary slice. FDG-uptake of the allograft (A, aTX: 1.22 cpm/mm^2^/MBq) increased by nearly 7 times when compared to control (A, CTR: 0.18 cpm/mm^2^), while the FDG uptake in the syngeneic transplant (B, sTX: 0.26 cpm/mm^2^) was 1.52 times that of control (B, CTR: 0.17 cpm/mm^2^). FDG-uptake of the kidney with ATN (C, ATN: 0.19 cpm/mm^2^) was 1.58 times that of control (C, CTR: 0.12 cpm/mm^2^), while the FDG-uptake in the kidneys with acute CSA toxicity (D, left: 0.13 cpm/mm^2^, right: 0.14 cpm/mm^2^) was even lower. Notably, in ATN the FDG-uptake clearly emphasizes the outer medulla region.

Examination of inflamed tissue by autoradiography has previously shown that FDG-accumulation is correlated to cellular infiltrates in different pathophysiological scenarios [20]. Therefore, we have chosen autoradiography as a reference method to validate FDG-PET results. In each recipient, radioactivity of renal parenchyma (mean activity in cpm/mm^2^/MBq) of the reference kidney was compared to the radioactivity of the graft. POD 4 was chosen for autoradiography, because FDG-uptake in allografts peaked at this time point in PET, nonetheless the integrity of the graft was still intact as confirmed by histology (Figure 5A). On POD 4, allograft FDG-uptake was 7.42±1.61 times (n = 3, p<0.05 vs. CTR and sTX) higher than uptake of controls, whereas FDG-uptake in sTX, ATN, and acute CSA nephrotoxicity was not statistically different from CTR (sTX: 1.54±0.05, ATN: 1.59±0.11, CSA: 0.81±0.09 times of control, n = 3). An exemplary measurement is shown in Figure 6.

The rat kidney consists of renal pelvis (7.3±0.6%), medulla (15.8±0.5%) and cortex (76.9±0.9%) as confirmed by micro-dissection (n = 14). In PET, FDG-uptake of renal parenchyma was measured with exclusion of renal pelvis. Therefore, theoretically 17.1±0.6% of the signal measured by PET can be attributed to the medulla and 82.9±0.6% to the cortex. Hence, the key question was still, if the FDG-uptake of medulla and cortex equals. Discriminating medulla and cortex for certain can only be performed, if visually confirmed. Therefore, we have chosen to investigate FDG-uptake of medulla and cortex by autoradiography. In native kidneys and syngeneic grafts, the renal medulla possesses an FDG-uptake, which is 1.88±0.18 (CTR) or 1.98±0.08 (sTX) times higher than the matching renal cortex, respectively (Table 1). Therefore, 72.1±0.8% of PET signal can be taken as a signal of renal cortex and 27.9±0.8% of renal medulla.

**Table 1:** FDG-uptake of renal medulla and cortex (mean±SEM).

	Control (n = 9)	ATN (n = 3)	CSA (n = 3)	sTX (n = 3)	aTX (n = 3)
Cortex [cpm/mm^2^/MBq]	0.12±0.01	0.21±0.01	0.11±0.02	0.22±0.03	0.90±0.16 *
Medulla [cpm/mm^2^/MBq]	0.23±0.02	0.36±0.05	0.17±0.01	0.44±0.07	1.26±0.25 *
MC-ratio	1.91±0.12	1.73±0.15	1.59±0.13	1.98±0.08	1.39±0.08
Average activity in times of control		1.59±0.11	0.81±0.09	1.54±0.05	7.42±1.61 *

FDG-uptake (mean±SEM, * p<0.05 vs. control and vs. sTX) of renal medulla and cortex was assessed by autoradiography. ATN: acute tubular necrosis, sTX: syngeneic transplant, aTX: allogeneic transplant, MC-ratio: medulla/cortex-ratio, average: ((cortex*3)+medulla)/4.

The FDG-ratio of medulla and cortex (MC-ratio) changes in kidneys undergoing AR. In contrast to graft medulla, the cortex of aTX revealed a distinct higher FDG-uptake than the renal cortex of CTR and sTX. Therefore, the MC-ratio of aTX dropped to 1.39±0.08. As confirmed by HE-staining (data not shown), a lower ratio was linked to a more intense infiltrate in the cortex than in the medulla. In summary, we found that areas with increased FDG-uptake coincide with areas of inflammatory cell infiltrate.

### Histology

Signs of acute rejection are glomerulitis, tubulitis, endothelialitis, and graft infiltration [12], [21]. During allograft rejection, leukocytes are recruited into the organ [16], [17]. These highly activated leukocytes accumulate FDG, which can be quantified by PET [18]. Starting from POD 1, we found marked glomerulitis, tubulitis, endothelialitis, and graft infiltration in the aTX group (Table 2, Figure 5A). Signs of acute rejection increased until POD 4. Nevertheless, the integrity of the graft was still preserved. In contrast, graft recovery on POD 7 revealed a loss of integrity due to necrosis and hemorrhage (Figure 5A). As demonstrated (Figure 5, Table 2), no histological signs of rejection were found in controls (12±1 leukocytes/field of view (FOV), ti0 (total interstitial inflammation score, Table 2), n = 18) or sTX (26±4 leukocytes/FOV, ti0, n = 6). Allograft infiltration was already existent on POD 1 (Figure 5B, 77±2 leukocytes/FOV, p<0.05 vs. sTX and CTR, ti1–2, n = 6), aggravated on POD 2 (98±12 leukocytes/FOV, ti2–3, n = 6), peaked on POD 4 (142±4 leukocytes/ FOV, ti3, n = 6), and decreased slightly on POD 7 (115±15 leukocytes/FOV, ti3, n = 6). Primary renal morphologic lesions in CSA treated animals included proximal tubule collapse and vacuolization and, less frequently, interstitial edema and vacuolization of interstitial cells (POD 4, leukocytes: 7±1/FOV, n = 6). Kidneys with ischemia/reperfusion damage presented histological signs of ATN, i.e., tubular dilation, swelling, and necrosis, in addition to intraluminal brush border debris, protein casts and marginal infiltration (leukocytes: 17±3/FOV, n = 3) .

**Table 2:** ti-score of control kidneys, syngeneic, and allogeneic grafts.

Group	ti-score	Quantitative criteria for cellular interstitial inflammation
CTR (18), sTX (6)	ti0	no or trivial interstitial inflammation (<10% of parenchyma)
aTX POD 1 (4)	ti1	10–25% of parenchyma inflamed
aTX POD 1 (2), 2 (3)	ti2	26–50% of parenchyma inflamed
aTX POD 2 (3), 4 (6), and 7 (6)	ti3	>50% of parenchyma inflamed

POD: post operative day; number of samples in brackets, ti-score according to [21].

### Correlation of FDG-uptake in renal parenchyma to the inflammatory infiltrate

**Figure 7:**
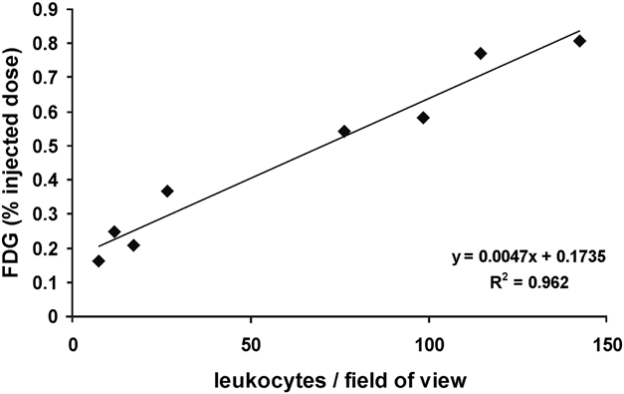
Correlation of the infiltrating leukocytes (as evaluated by light microscopy) and of the FDG-uptake (%ID as assessed by PET) in different groups of kidneys. A significant correlation of leukocytes and FDG signal was found (r^2^ = 0.96).

To verify our hypothesis that the uptake of FDG (%ID) is directly related to the degree of inflammatory infiltration and thereby to the grade of AR, we correlated the leukocytes/FOV of each group (aTX POD 1, 2, 4, 7, sTX, ATN, CSA-induced nephrotoxicity, and native controls) to their according %ID (Figure 7). This correlation was found strongly significant (R^2^ = 0.96).

### How accurate is FDG-PET for the diagnosis of AR in rat?

**Figure 8:**
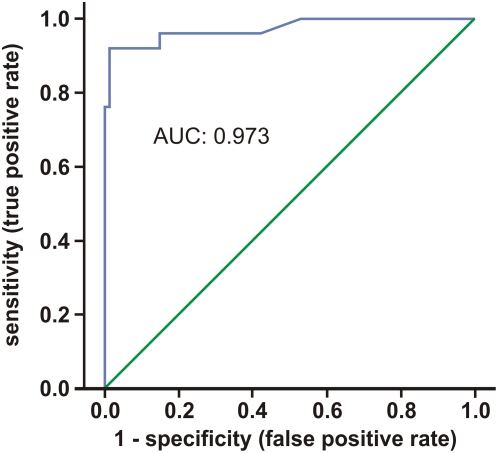
Receiver Operator Curve for diagnostics of acute rat renal allograft rejection by FDG-PET. The curve is the regression line that summarizes the overall diagnostic accuracy. A perfect prediction would yield AUC = 1 (area under the curve), whereas AUC = 0.5 would suggest predictive accuracy equal to that of chance alone. Thus, the area measures discrimination that is, the ability of the test to correctly classify those with and without the disease. AUC was 0.973.

We evaluated the ability of FDG-PET to discriminate between kidneys from different control models (sTX, CSA-toxicity, ATN, and native controls) and individuals with acute allograft rejection by a Receiver Operating Characteristic (ROC) curve. Figure 8 shows the corresponding ROC curve and area under the curve (AUC). The AUC was 0.973. Specificity (true negative rate) and sensitivity of FDG-PET for detection of AR were calculated (Table 3) and with 98.8% and 92.0%, respectively, found also very high in our model.

**Table 3:** The four outcomes in a contingency table.

		acute rejection (histology)		n
		positive (n)	negative (n)	
FDG-PET (FDG cut-off value: 0.36% ID)	positive (n)	23	1	24
	negative (n)	2	80	82
n		25	81	106

ID: injected dose; true positive rate: 0.92 (23 out of 25); false positive rate: 0.01 (1 out of 81).

## Discussion

In this study, we present high resolution small animal FDG-PET as a new approach for non-invasive imaging of acute renal allograft rejection in rats. We found a significant correlation of the entirely image-based measurement of *enhanced* FDG-uptake (cpm/mm^2^/MBq and %ID) in transplants undergoing rejection to reference methods, i.e., to the histological quantification of infiltrating leukocytes (Figure 5B and 7). In contrast, native controls lacking signs of acute rejection in histological examination (Figure 5A), showed only low, *baseline* FDG-uptake which correlated significant with the histological examination (Figure 7). In addition, histological evaluation of syngeneic transplants did not reveal significant glomerulitis, tubulitis, endothelialitis, and only marginal graft infiltration (ti0) (Figure 5, Table 2). This marginal infiltration may be attributed to transplantation-associated ischemia-reperfusion damage [22]. Rats with acute CSA nephrotoxicity or kidneys with histological signs of ATN did show neither significant elevation in FDG-uptake nor extended infiltration with leukocytes. In case of ATN this might be due to the fact that the infiltration observed emphasizes the outer medulla (as seen in histology and autoradiography) which contributes to a small amount only to the total renal FDG-uptake. In autoradiography, a second reference method used, the FDG-uptake of allogeneic transplants was distinctly higher than the uptake of kidneys with CSA toxicity, ATN, native controls or sTX (Figure 6, Table 1).

To evaluate diagnostic tests, ROC curves and AUC can be calculated [23]. The best possible prediction method would yield a point in the upper left corner or coordinate (0/1) of the ROC space, representing 100% sensitivity (no false negatives) and 100% specificity (no false positives). Interpretation of the AUC is easier: the higher the AUC, the better, with 0.5 indicating random performance and 1.0 denoting perfect performance. Figure 8 shows the corresponding ROC curve; the area under the curve was 0.973, indicating a high overall accuracy. In order to provide an optimal diagnostic cut-off value for the FDG-PET, the Euclidean distance was calculated. We propose a graft value of 0.36% ID FDG, which seems to be the optimal compromise between sensitivity and specificity.

Drainage of FDG into the renal pelvis might be a problem when assessing FDG-uptake in the renal parenchyma. Therefore, we have chosen late acquisitions three hours after injection to reduce the instantaneous amount of tracer in the urine during the PET scan. The time interval for acquisition was defined according to results of dynamic FDG-investigations which showed a very low and stable renal FDG-uptake after 180 min p.i.. An impact of renal function on FDG-uptake was excluded by comparison of renal fluoride clearance to FDG-uptake, which did not correlate (Figure 4) [24].

It is of note, that not simply the average activity of renal parenchyma revealed discrepancies between rejected and kidneys not undergoing rejection. Controls possess a certain medulla to cortex (MC) ratio of FDG-uptake. This MC-ratio was identical to the MC-ratio of syngeneic transplants, but significantly decreased in rejected allografts (Table 1). One reason for these differences might be the accentuation of the rejection-related infiltrate in the renal cortex as observed in histology [25]. Verification of our hypothesis that the MC-ratio changes in AR by small animal PET failed due to small volumes of rat kidneys and resolution limitations. We were regrettably unable to differentiate the activity of renal parenchyma for certain into medulla- and cortex-related tissue-activity. Nevertheless, our observation might be useful in PET-based detection of AR in humans, since the MC-ratio could be easily calculated by defining and measuring two volumes of interest in a kidney transplant: one volume (medulla) placed distal of the renal pelvis, the other next to the borders of the kidney (cortex). This should be evaluated in future studies in humans.

Functional imaging with FDG is widely used (and therefore available in common) in clinical setups, e.g., diagnosis and staging of malignant disease, in image-guided therapy planning, and detection and grading of inflammation, be it infectious, autoimmune, or of other origin [26], [27]. On one hand, FDG-uptake is specific and unique for detection of enhanced metabolic activity. On the other hand, metabolic activation, and therefore FDG-uptake, is not disease-specific. Thus, graft infection or tumors might cause similar FDG-uptake in PET. Nevertheless, if clinical symptoms, pointing to malignancy (weight loss, night sweat, etc.), or to urinary tract infection (fever, dysuria, etc.), are present target-orientated, additional diagnostics, like ultrasound, blood or urine tests, should be applied.

One might worry about the exposure to radiation when applied to humans, but the dosage applied, is rather low and close to other clinical investigation techniques, e.g., lower than the dosage which is applied during an abdominal computer tomography. In addition and in contrast to several contrast media used in computer tomography or magnetic resonance imaging, the tracer is neither toxic to kidney nor carries the risk of nephrogenic systemic fibrosis.

A relevant diagnostic problem in case of graft dysfunction might be the discrimination of AR and CAF. CAF also featuring a certain degree of allograft infiltration is the most common reason for graft loss [6], [7], [12]. In this case, the time point of occurrence of the symptoms, and the kinetic of the process might be helpful. According to the Banff-criteria and in contrast to AR, the kinetic of CAF is rather slow, its degree lower, and besides, its typical histological infiltration pattern rather nodular than diffuse [12]. This might offer a diagnostic pattern in PET analysis. Graft FDG-PET may also be useful in monitoring therapy effects, for instance, illustrating the impact of immunosuppressive therapy escalation.

In conclusion, we present and validate a non-invasive, entirely image-based method to assess acute renal rejection using FDG-PET. We suggest that this method may have a potential clinical impact for the diagnosis of AR and may be useful to discriminate AR from ATN and acute CSA-induced nephrotoxicity, in patients with severe contraindications to perform graft biopsy.

## Materials and Methods

### Animal models

Male Lewis–Brown-Norway (LBN) and Lewis (LEW) rats (270–330 g, Charles River, Sulzfeld, Germany) with free access to standard rat chow (Ssniff, Soest, Germany) and tap water were used. Experiments were approved by a governmental-committee on animal welfare (Landesamt für Natur, Umwelt und Verbraucherschutz Nordrhein-Westfalen) and were performed in accordance with national animal protection guidelines. Surgeries were performed under anesthesia with ketamine 100 mg/kg body weight intra peritoneal (i.p.) and xylazine 5 mg/kg BW i.p. (CEVA Tiergesundheit, Düsseldorf, Germany). Further doses of ketamine were injected as needed. Transplantation was simultaneously performed by two investigators as published before [28]–[30]. In short, the left kidney including ureter, renal artery, a piece of aorta, and renal vein were transferred into the recipient. Kidneys from age- and weight-matched LBN were transplanted into LEW (aTX, n = 24) or LBN (sTX, n = 6) without immunosuppression. Transplantations were performed immediately after left nephrectomy of the recipient. While the total operation time of the recipient did not exceed 90 min, the ischemia time of the graft was always shorter than 40 min. The chosen aTX model leads to histological and functional changes typical for acute rejection [29], [31]. Grafts and control kidneys were recovered on day 1, 2, 4, or 7 after transplantation. ATN and acute CSA toxicity were induced in a modification according to Kim et al.[32]. Ischemia/reperfusion injury model (ATN, n = 3) was prepared by temporary ligation of the left renal artery for 40 minutes. Renal artery was dissected as in TX-groups and ligated using a microvascular clamp. After clamp release, the returning of original surface colour of the kidneys was confirmed visually. For acute CSA-induced nephrotoxicity, rats were treated with 50 mg/kg cyclosporine A (Sandimmun, Novartis, Nürnberg, Germany) i.p. for two days before PET-measurements and kidney recovery on day 4 (n = 3).

### Image acquisition – PET

Clinical grade ^18^F-fluoride and FDG were produced on site using an RDS 111 cyclotron (CTI, Knoxville, TE, USA). The FDG-uptake was calculated from a whole body acquisition of 30 min length in a high-resolution small animal PET scanner 3 hours after i.v. injection of 30 MBq FDG in 100 µl 0.9% NaCl solution in a tail vein.

Afterwards the catheter was purged with additional 900 µl 0.9% NaCl solution. The animals remained in a restrainer without anaesthesia until start of the scan and were hydrated by i.v. injection of 1000 µl of 0.9% NaCl solution hourly. Acquisition started 3 h after FDG-injection to reduce tracer uptake in the kidneys caused by renal excretion of FDG.

During acquisition, rats were anaesthetized with oxygen/isoflurane inhalation (2% isoflurane, 0.7 l/min oxygen) and body temperature was maintained at physiological values by a heating pad. PET scans were performed with the high-resolution multi-wire chamber-based animal PET camera quadHIDAC (Oxford Positron Systems Ltd, Oxford, UK). The field of view (FOV) of the PET scanner is 28 cm axially and 17 cm in diameter. The spatial resolution of the PET scanner is <1.0 mm and is constant over the whole FOV [33].

The FDG-PET acquisition was followed by i.v. injection of 5 MBq ^18^F-fluoride without motion of the position of the rat in the scanner. Another PET acquisition of 15 min length was immediately initiated after ^18^F-fluoride injection for identification of renal parenchyma (volume of interest, VOI) or of 60 min for measurement of fluoride clearance.

### Analysis of PET images and quantitative evaluation

#### FDG-uptake

A renal parenchyma VOI manually traced around the kidneys on reconstructed images 2 min after ^18^F-fluoride injection was transferred to the FDG images. The renal pelvis was carefully excluded from the VOI. Mean FDG-uptake of the renal parenchyma was calculated by the ratio of total counts and volume.

#### Fluoride Clearance

Renal fluoride clearance and spilt renal function was calculated by the ratio of the renal excreted activity 60 min p.i. and the integral of the blood pool time activity curve in a subgroup of acquisitions (n = 22) as recently described [24].

#### High-resolution micro-autoradiography

To validate the signal obtained by PET animals were euthanized and the kidneys were harvested immediately after FDG-scanning on POD 4. High-resolution micro-autoradiography (μ-imager, Biospace Measures, Paris, France) was performed on cryosections (10 µm thick) of snap-frozen transplant or normal kidney. Two regions of interest were visually identified and mean activity (cpm/mm^2^) was measured for 3 h. One region was defined as the medulla-region, the other as the cortex-region. A ratio medulla/cortex (MC-ratio) was calculated. Additionally, a more “realistic” mean whole kidney-radioactivity (parenchyma: 75% cortex, 25% medulla), was calculated as followed: (mean activity of medulla [cpm/mm^2^/MBq+mean activity of cortex [cpm/mm^2^/MBq] * 3)/4.

### Histology

Portions of kidneys were snap-frozen and fixed in 4% formaldehyde in PBS. Histological changes (glomerulitis, tubulitis, endothelialitis, and infiltration) were examined by light microscopy in paraffin-embedded tissue with hematoxylin–eosin (HE) staining. Graft infiltration was quantified according to the ti-score (total interstitial inflammation score, Table 2), which was recently added to the Banff classification [21]. The basic concept of the Banff classification is that the amount of the interstitial infiltrate and the severity of the tubulitis indicate the grade of cellular rejection. Infiltrating cells were determined by counting and averaging the number of leukocytes present within 10 fields of view (FOV, 350×250 microns each) in the renal cortex. Cortex was chosen, because quantification of medullary inflammation is not a reliable indicator of AR [25].

### ROC Curve

The joint distribution of true positive and true negative rates was summarized as a ROC curve (Figure 8), which is used to evaluate diagnostic tests [23]. All ROC curves begin in the bottom-left corner and rise to the top-right corner. Moving along the ROC curve represents trading off false positives for false negatives. The calculated regression curve indicates the distribution of the paired sensitivity and specificity values. The AUC represents an analytical summary of test performance and displays the trade-off between sensitivity and specificity.

### Statistics

Data was analyzed by the help of standard software (SPSS 13.0). Laboratory data was compared with ANOVA variance analysis for multiple comparisons where appropriate. Data is presented as mean values±SEM (n = number of rats, samples, or experiments). Significance was inferred at the p<0.05 level.
